# Research Hotspots and Trends in Music Therapy Intervention for Patients With Dementia: A Bibliometrics and Visual Analysis of Papers Published From 2010 to 2021

**DOI:** 10.3389/fpsyt.2022.860758

**Published:** 2022-04-28

**Authors:** Shao Yin, Fengya Zhu, Zhao Li, Deya Che, Liuying Li, Lu Zhang, Yue Zhong, Biao Luo, Xiaohan Wu

**Affiliations:** ^1^Hospital of Chengdu University of Traditional Chinese Medicine, Chengdu, China; ^2^Zigong First People's Hospital, Zigong, China; ^3^Department of Medical Information Engineering, Chengdu University of Traditional Chinese Medicine, Chengdu, China

**Keywords:** dementia, music therapy, research hotspots, bibliometric analysis, biclustering analysis, CiteSpace, Web of Science

## Abstract

**Background:**

As a serious public health problem, dementia has placed a heavy burden on society and families. Evidence suggests that the use of music therapy as a non-pharmacological intervention has certain advantages with respect to reducing the behavioral and psychological symptoms of dementia (BPSD) and improving the cognition and mental status of dementia patients. However, research trends and hotspots regarding music therapy intervention for dementia analysis have not been systematically studied *via* bibliometric analysis.

**Methods:**

We searched the Web of Science Core Collection (WoSCC) for texts published between January 1, 2010, and October 31, 2021, and visualized country, institution, journal, keyword co-occurrence, keyword emergence and keyword clustering.

**Results:**

A total of 217 articles from the WoSCC database were analyzed. In this research field, the annual number of publications has generally shown a slowly increasing trend, and the United States has the most publications and the most frequent cooperation among countries. University College London (UCL) has the most extensive influence among research institutions. Among articles, those published in the JOURNAL OF ALZHEIMER'S DISEASE were the most numerous, with 20 such articles being published, accounting for 9.22% (20/217) of the total. Comprehensive analysis of five clusters *via* biclustering shows that the research hotspots in this field during the past 11 years have mainly focused on the autobiographical memory, cognitive function, mental state and BPSD of dementia patients.

**Conclusion:**

This study conducted a bibliometric and visual analysis of relevant studies concerning music therapy intervention for dementia patients. Psychological problems faced by dementia patients and the topics of quality of life, individualized music therapy, the mental state of caregivers and other related topics may be important research directions in the future. Therefore, the question of how to develop standardized research protocols and identify unified efficacy evaluation indicators should be a focus of and difficulty for future research.

## Introduction

According to a survey by the World Health Organization in 2021, more than 55 million people worldwide suffer from dementia, a number which is rising at a rate of 10 million new cases per year (1). The number of dementia patients worldwide is expected to exceed 150 million by 2050, and the cost of treatment is expected to double by 2030 (2). As the country with the largest number of dementia patients, China accounts for approximately one quarter of global prevalence of dementia (3). Clearly, dementia has become a serious public health problem, placing a heavy burden on society and families. Alzheimer's disease (AD), as the main type of dementia, leads to a loss of function and cognitive and behavioral issues in patients (4), and 90% of dementia patients exhibit behavioral and psychological symptoms of dementia (BPSD), which become manifest as excitement, sleep disorder, anxiety and aggressive behavior (5). These symptoms reduce the quality of life of patients and caregivers (6), increase the cost of care, and entail severe challenges for family caregivers (7), even leading to a deterioration of the relationship between family members (8).

Management of BPSD, cognition and mental status in dementia patients is mainly divided into drug intervention and non-pharmacological intervention categories (9). However, drug interventions are often accompanied by adverse reactions, increasing the risk of death among patients (10), and may lead to cognitive decline and an increase in the number of falls among patients (11). The safety and effectiveness of drug interventions remain controversial (12). Compared with drug interventions, non-pharmacological interventions are more effective in improving cognition and reducing BPSD in dementia patients (13, 14). A number of studies have shown that non-pharmacological intervention has a low level of risk, can effectively manage BPSD symptoms in dementia patients (15), and can accomplish personalized nursing interventions according to the situation of patients and their families (16), which can improve patient quality of life and enhance caregiver satisfaction (17), so this approach is a scientific and effective nursing intervention.

As an important intervention in the non-pharmacological treatment of dementia, music therapy is mainly divided into the categories of active music therapy and passive music therapy (18), that is, active participation in singing, instrument playing and music creation, and passive listening to music. As a nonverbal therapy (19), music therapy is not affected by the course of the disease and can be used at all stages of dementia (20). Participating in singing activities can effectively reduce patients' excited-aggressive moods (21), improve their quality of life, reduce pressure on caregivers, and promote the happiness of family members (22). Personalized music therapy based on patients' musical memory can reduce BPSD (23, 24). A study of 60 patients with moderate to severe dementia who received music therapy intervention reported improvement in psychiatric symptoms and reduced frequency and severity of BPSD (25). Evidence shows that music therapy seems to be effective, but there is no systematic and comprehensive report concerning visual analysis of research hotspots and development trends in music therapy regarding the non-pharmacological management of dementia.

Publications have shown that music therapy appears to be effective in the intervention of dementia. However, as far as we know, no one has systematically analyzed the subject of this field by means of bibliometric analysis. Therefore, this study aims to research hotspots and trends in music therapy intervention for patients with dementia from 2010 to 2021 through bibliometrics. As an effective statistical method, bibliometrics and visual analysis can not only evaluate the published literature but also predict development trends in this field, thus providing a relevant basis for subsequent studies (26). Therefore, based on the WoSCC database, this study used the CiteSpace and gCLUTO software to examine trends and co-occurrence matrices, aiming to analyze the research status, hotspots and development trends in this field, help relevant researchers strengthen their understanding of this field and promote the benign development of relevant research in this field.

## Materials and Methods

### Data Collection and Search Strategy

A search of the Web of Science Core Collection (WoSCC) database for studies published between January 1, 2010, and October 31, 2021, regardless of language, was performed by using the following search parameters: [TS = (music Therapy) OR TS = (music)] AND TS = (dementia).

*Inclusion criteria*: 1. Journal articles concerning music therapy intervention in dementia; 2. Publication period between January 1, 2010 and October 31, 2021; 3. Article type is ‘Article’.

*Exclusion criteria*: 1. Review, conference paper, letter, comment, etc.; 2. Duplicate literature.

**Figure 1:**
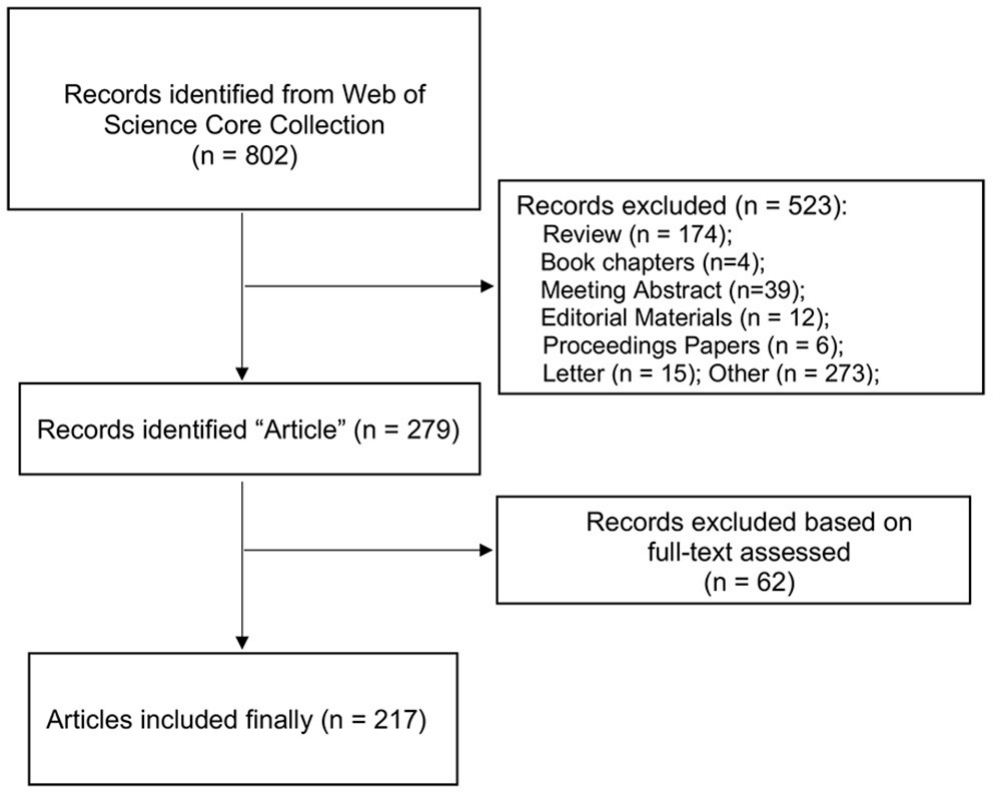
Flowchart of included and excluded studies.

Two independent reviewers (SY and ZL) conducted a search according to the retrieval strategy, filtered the articles according to the inclusion and exclusion criteria, imported the qualified literature into Endnote (X9.1) in an appropriate format for subsequent operations, performed a cross-check between the two reviewers, and consulted a third reviewer (FYZ) concerning any discrepancies. To reduce deviation, the search and data extraction were completed on November 6, 2021, and the flow chart is shown in Figure 1.

### Data Analysis

#### Data Extraction and Network Analysis

Employing the basic characteristics of the WoSCC database, number of papers, publication year and publication country were extracted and summarized, and then a line chart, national literature volume map and international cooperation map were drawn. Then, we imported the data into Citespase 5.8. R1 in refworks format, set the time parameters to between January 1, 2010 and October 31, 2021, year per slice = 1, and set other parameters to the default values, adjusting as needed to draw the network hotspot map.

#### Keywords Biclustering Analysis

The gCLUTO software (version 1.0, Graphical CLUstering Toolkit, a graphical front-end for the CLUTO data clustering library developed by Rasmussen, Newman, and Karypis from the University of Minnesota) (27) was used to conduct a biclustering analysis of high-frequency keywords. First, qualified literature from the WoSCC database was imported into Bibliographic Item Co-Occurrence Matrix Builder (BICOMB, version 2.0, designed by Professor Lei Cui from China Medical University) to construct a co-occurrence matrix of high-frequency keywords, and then a cluster analysis was conducted using gCLUTO, clustering method will use the repeated bisection, *I*^2^ as the optimization function, and cosine function as the similarity coefficient, and set other parameters to the default values, to generate a matrix visualization and a mountain visualization to show the relationship between high-frequency keywords and the source literature as a means of identifying research hotspots related to music therapy interventions for patients with dementia.

## Results

### Publication Years and Country Distribution

**Figure 2:**
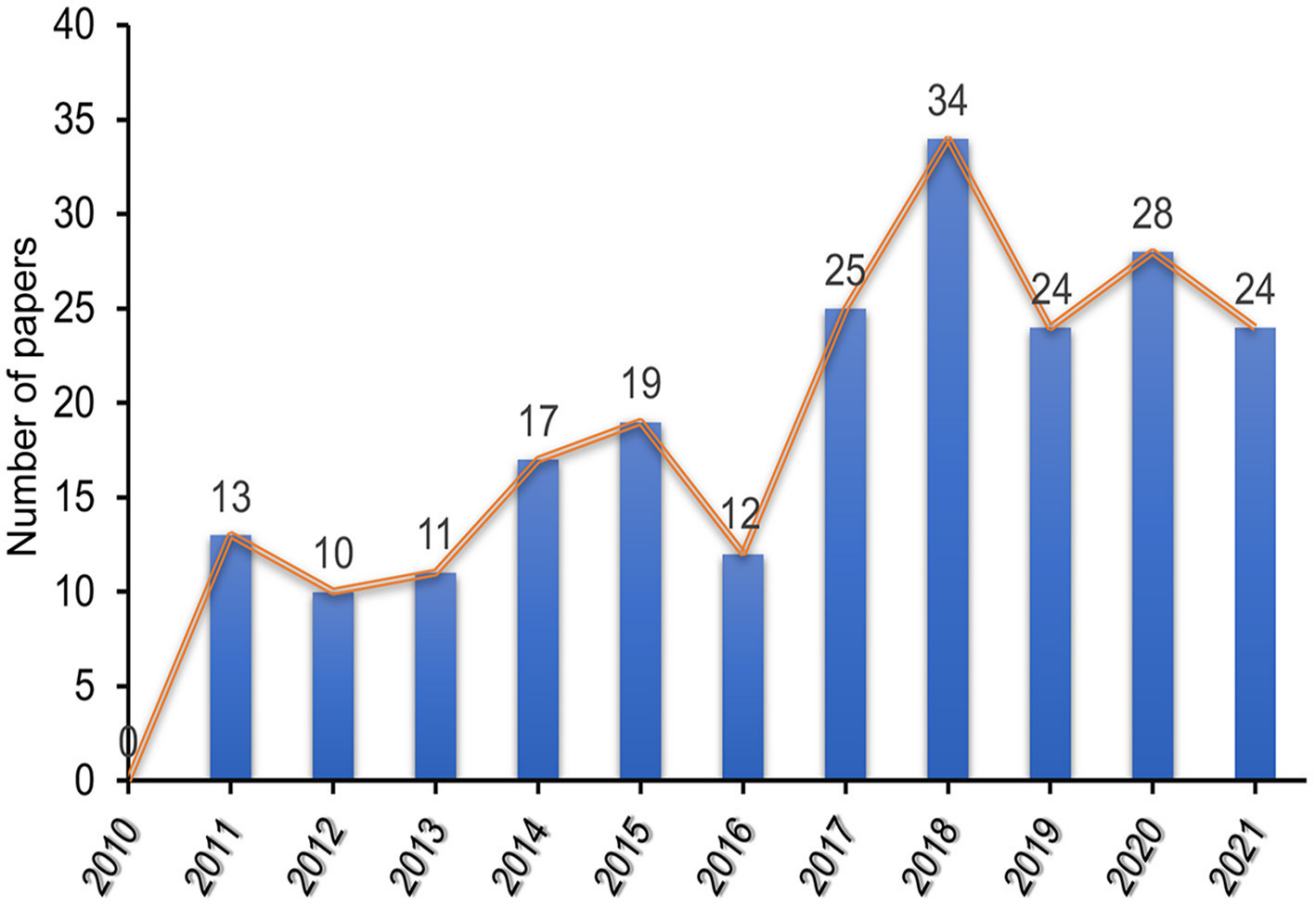
Annual trend chart of publications included in WoS database from 2010 to 2021.

**Figure 3:**
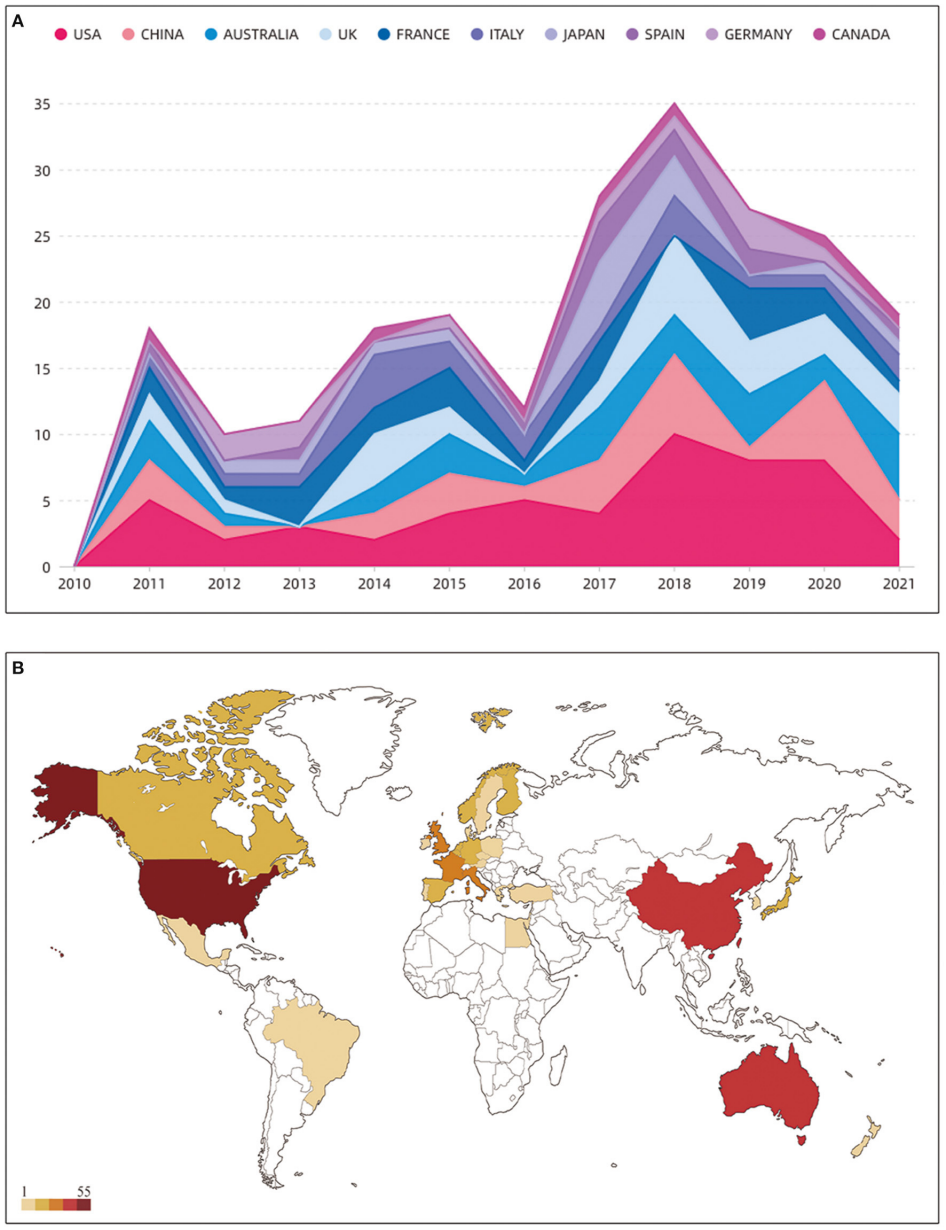
**(A)** The growth trends of the top 10 countries in terms of the number of published articles from 2010 to 2021. **(B)** Geographical distribution of music therapy for dementia publications.

As an important index, changes in the number of articles can directly reflect research trends in the use of music therapy as an intervention for dementia. After screening, a total of 217 qualified articles were included, as shown in Figure 1. The results in Figure 2 show that the overall number of articles in this research field was low between 2010 and 2021 and that research began to appear and increase slowly in 2011, with the largest number of articles being published in 2018 (34 articles). Although there was a slight decline in 2016 (12 articles) and 2019 (24 articles), the overall annual number of articles showed a slow upward trend. The summary results of annual publications from the top 10 countries in terms of the number of published articles between 2010 and 2021 (Figure 3A) showed a similar trend to that of overall publications in this field. However, in 2020, Figure 2 shows an increase in the number of published articles, while Figure 3A shows the opposite result, indicating that the emerging countries contributed to the number of publications in this field gradually increased and that this trend had a certain degree of influence.

**Figure 4:**
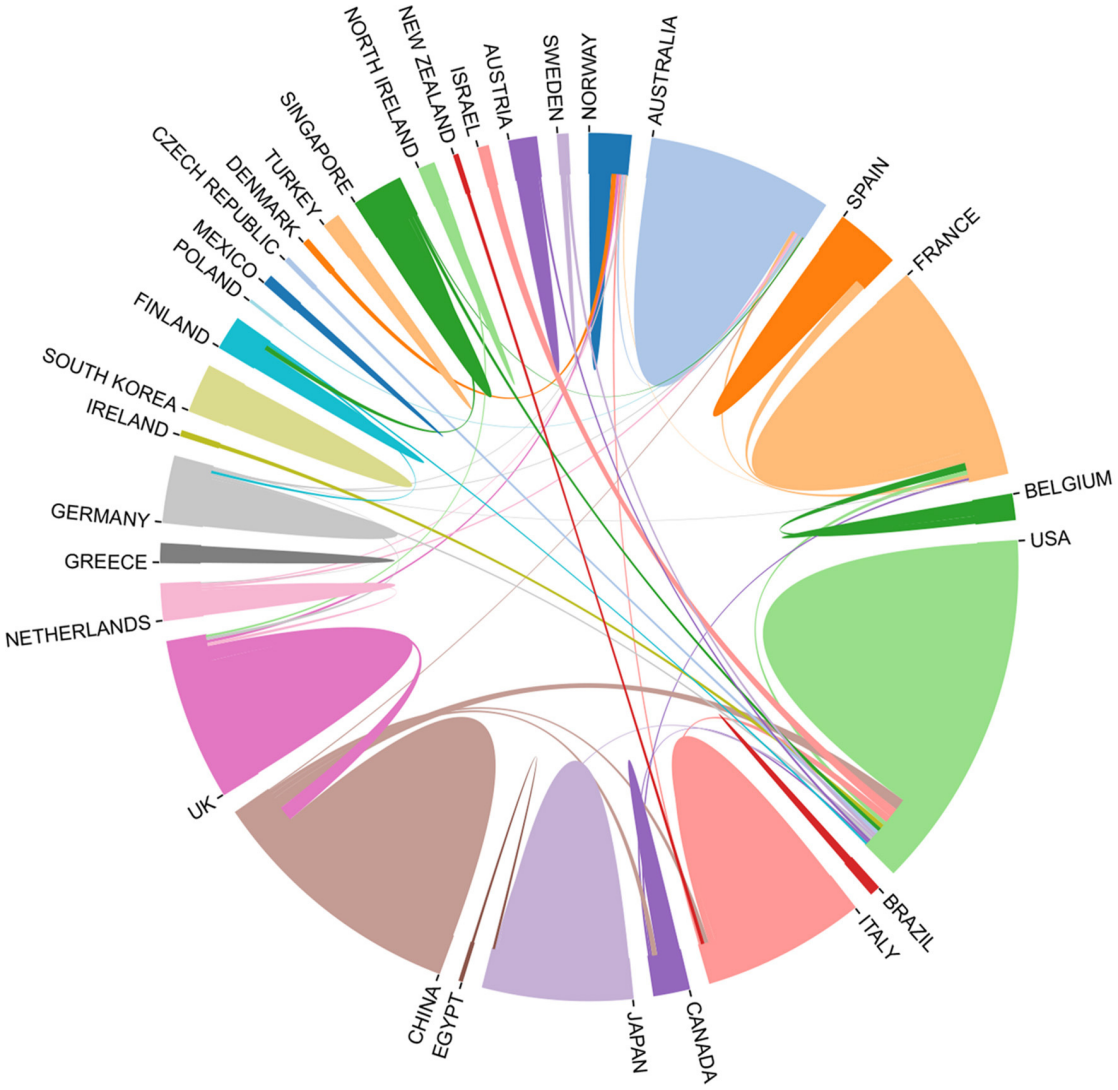
The cooperation of countries in the field of music therapy for dementia from 2010 to 2021.

A total of 30 countries have contributed to scientific research concerning the use of music therapy for dementia (Figure 3B), and cooperation among countries in this field is shown in Figure 4. After using CiteSpace to analyze country nodes, the top 10 countries in terms of number of publications and center value between 2010 and 2021 are presented in Table 1, in which center value is positively correlated with number of cooperative relationships. The top 3 countries in terms of the number of publications are the USA, China and Australia. Concerning countries that had a central value > 0.5 after selection, the analysis results show that the USA (publications: 53, centrality: 0.93), UK (publications: 27, centrality: 0.89), Italy (publications: 19, centrality: 0.65), New Zealand (publications: 2, centrality: 0.59) and the Netherlands (publications: 5, centrality: 0.59) have a high degree of influence in the field. Among these countries, the United States has outstanding advantages in this field, having produced the largest number of publications, and has cooperated with most countries in this field. It is worth noting that New Zealand ranks 21st in publication volume (publication: 2) but 4th in centrality (centrality: 0.89), which indicates that although New Zealand published few articles, the country has close relations and cooperation with other countries in this field.

**Table 1:** Top 10 countries and institutions in terms of number of publications and center value between 2010 and 2021.

**Items**	**Publication**			**Centrality**		
	**Ranking**	**Name**	**Number**	**Ranking**	**Name**	**Number**
Country	1	USA	53	1	USA	0.93
	2	China	30	2	UK	0.89
	3	Australia	28	3	Italy	0.65
	4	UK	27	4	New Zealand	0.59
	5	France	22	5	Netherlands	0.59
	6	Italy	19	6	China	0.49
	7	Japan	14	7	Denmark	0.30
	8	Spain	11	8	Canada	0.30
	9	Germany	11	9	Australia	0.21
	10	Canada	7	10	Norway	0.21
Institution	1	University College London	9	1	University College London	0.07
	2	Western Sydney University	5	2	Aalborg University	0.06
	3	Mie University	5	3	University of California, San Francisco	0.06
	4	University of Lille	5	4	University of Lille	0.04
	5	University of Paris Descartes (Paris 5)	4	5	The University of Texas at Austin	0.02
	6	University of California, San Francisco	4	6	University of California, Davis	0.02
	7	Macquarie University	4	7	University of Toronto	0.02
	8	University of Pavia	4	8	University of Pavia	0.01
	9	The University of Texas at Austin	4	9	Anglia Ruskin University	0.01
	10	Aalborg University	4	10	Western Sydney University	0.01

### Distribution of Issuing Institutions

**Figure 5:**
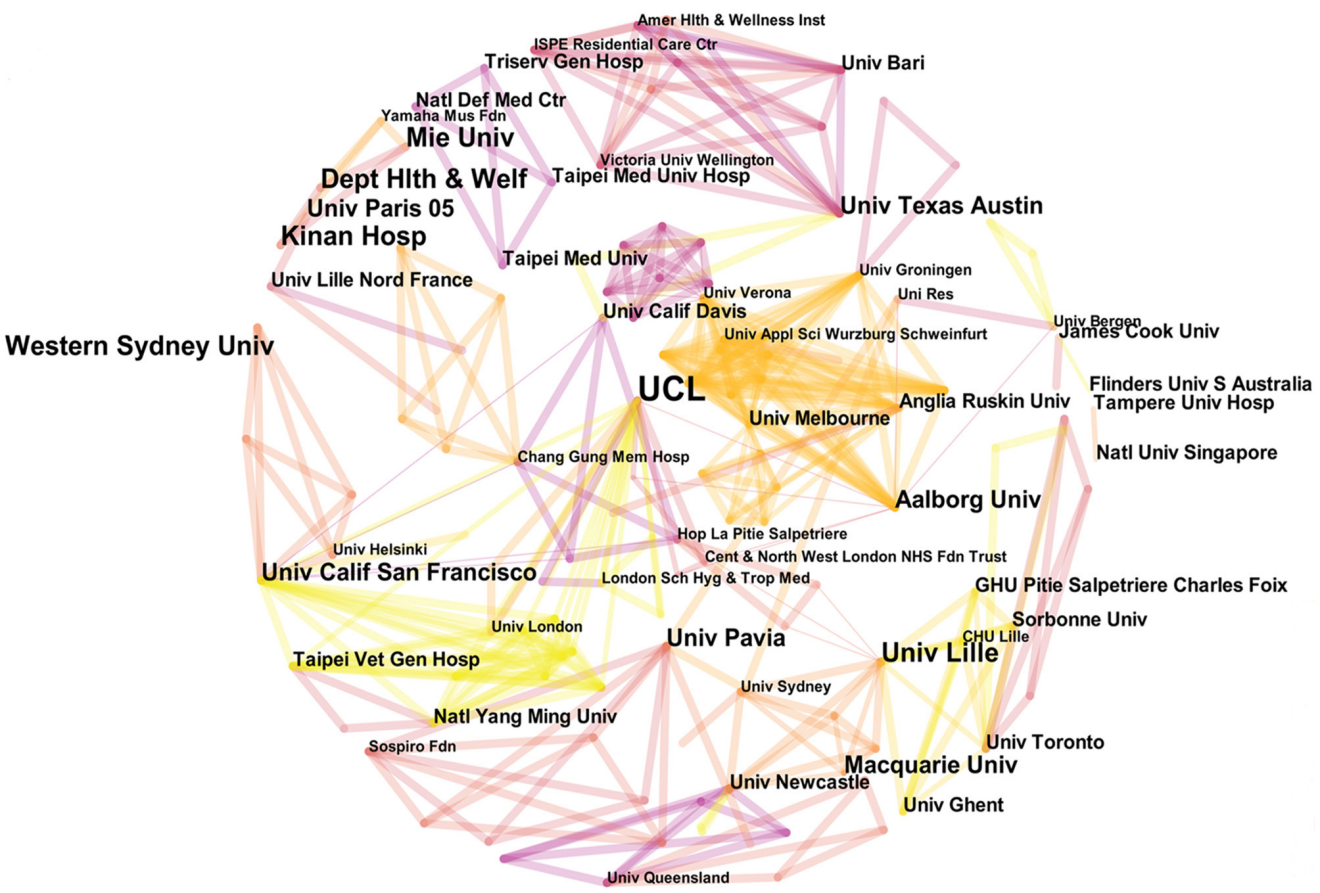
Academic collaboration between different institutions in the field of music therapy for dementia.

Institution nodes were analyzed by using CiteSpace, and a cooperative network diagram was constructed. Nodes represent institutions, and wires represent cooperation relationships between institutions. The generated cooperation network diagram contains 471 nodes and 880 wires, indicating that a certain degree of exchange and cooperation took place among institutions. The cooperation network can thus be seen, as shown in Figure 5. Table 1 lists the top 10 institutions according to number of publications and center values. The top 4 institutions with the largest number of publications are University College London (publications: 9), Western Sydney University (publications: 5), Mie University (publications: 5) and the University of Lille (publications: 5). Institutions that had a central value > 0.05 included University College London (publications: 9, centrality: 0.07), Aalborg University (publications: 4, centrality: 0.06) and the University of California, San Francisco (publications: 4, centrality: 0.06). According to comprehensive analysis of publication volume and central value, University College London is the main institution in this field and has the most extensive network of cooperative relationships.

### Most Active Journals

A total of 104 academic journals published articles concerning the use of music therapy for dementia. The top 10 journals published a total of 71 articles, accounting for 32.71% (71/217) of total records. Among these journals, the *JOURNAL OF ALZHEIMER'S DISEASE* has the highest number of publications, with a total of 20 articles, accounting for 9.22% of the total (20/217). We also summarize the citations, IF (2020), and WoS categories of these journals in Table 2, and these categories are basically divided into Geriatrics and Gerontology, Psychiatry, Neurosciences, and Nursing. It is worth noting that the most cited journal was also the *JOURNAL OF ALZHEIMER'S DISEASE* (IF = 4.472), which demonstrates its significant influence in the research field.

**Table 2:** Summary of the top 10 journals.

**Journal**	**Published numbers (%)**	**Total citations**	**IF 2020**	**WoS Categories**
Journal of Alzheimer's disease	20 (9.22%)	56	4.472	Neurosciences
Aging and mental health	11 (5.07%)	49	3.658	Geriatrics and gerontology; Psychiatry
International psychogeriatrics	6 (2.76%)	40	3.878	Psychology; Clinical geriatrics and gerontology; Gerontology psychiatry
Geriatric nursing	6 (2.76%)	4	2.361	Geriatrics and gerontology; Nursing
American journal of Alzheimer's disease and other dementias	5 (2.30%)	15	2.035	Geriatrics and gerontology; Clinical Neurology
Journal of clinical and experimental neuropsychology	5 (2.30%)	13	2.475	Psychology; Clinical; Neurology; Psychology
Geriatrics and gerontology international	5 (2.30%)	7	2.73	Geriatrics and gerontology
Frontiers in medicine	5 (2.30%)	2	5.093	Medicine; General and internal
International journal of geriatric psychiatry	4 (1.84%)	41	3.485	Geriatrics and gerontology; Psychiatry
Geriatrie et psychologie neuropsychiatrie de vieillissement	4 (1.84%)	5	0.838	Psychiatry; Psychology

### Analysis of Co-occurring Keywords

**Figure 6:**
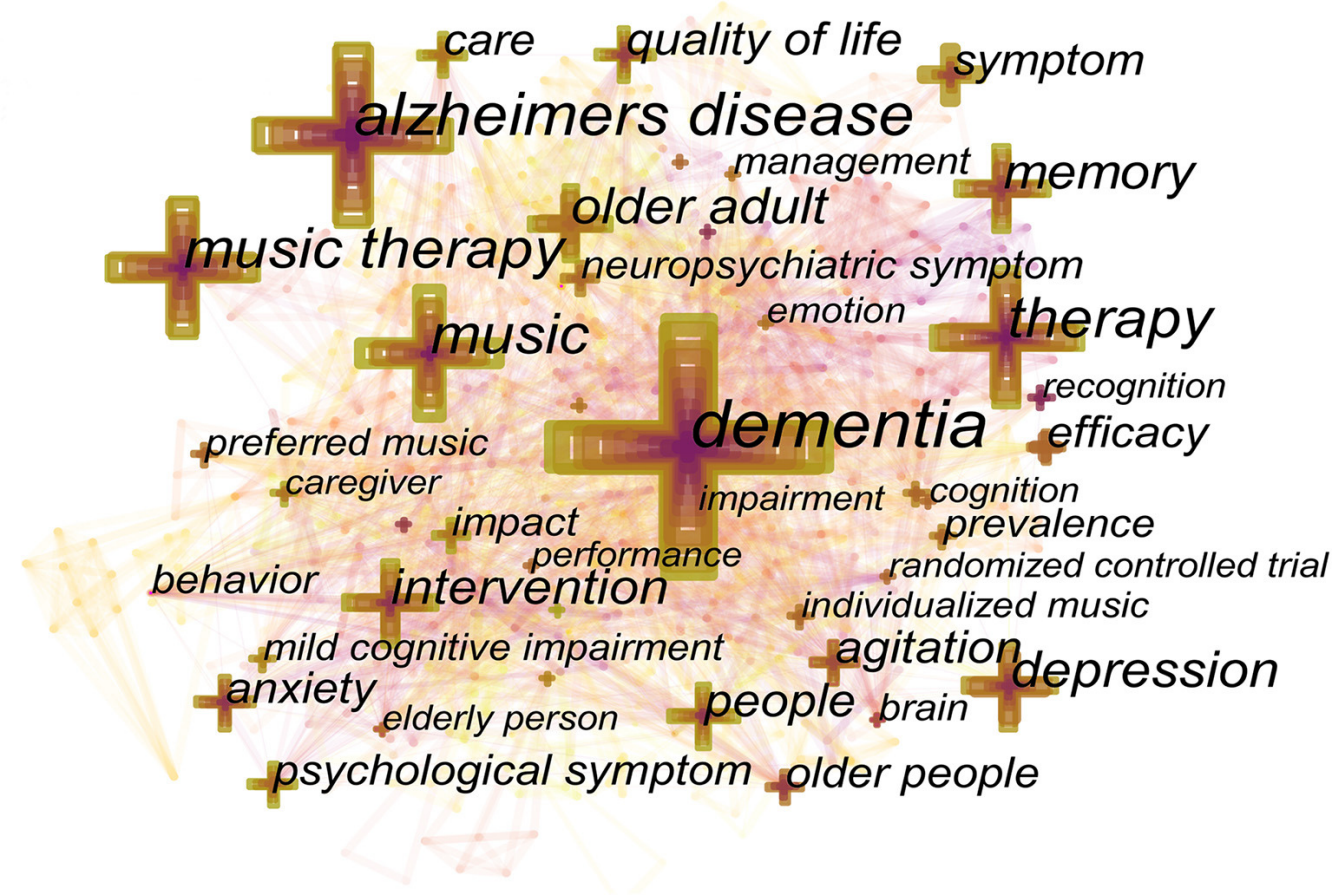
Keyword co-occurrence diagram.

Co-occurrence analysis of keywords can allow for a better understanding of research hotspots in this field. The keywords co-occurrence diagram is shown in Figure 6. Keywords with a high frequency in the 760 nodes include “dementia” (164 times), “Alzheimer's disease” (92 times), “music” (68 times), “music therapy” (67 times) and “older adult” (65 times). Keywords with higher central values include “behavior” (0.16), “agitation” (0.14), “elderly people” (0.10), “emotion” (0.09), “cognition” (0.07) and “caregiver” (0.05). High-frequency keywords are mainly based on different intervention measures and intervention groups, while high-central value keywords are closely related to the symptoms and nursing aspects of dementia patients.

### Research Frontiers Detection

**Figure 7:**
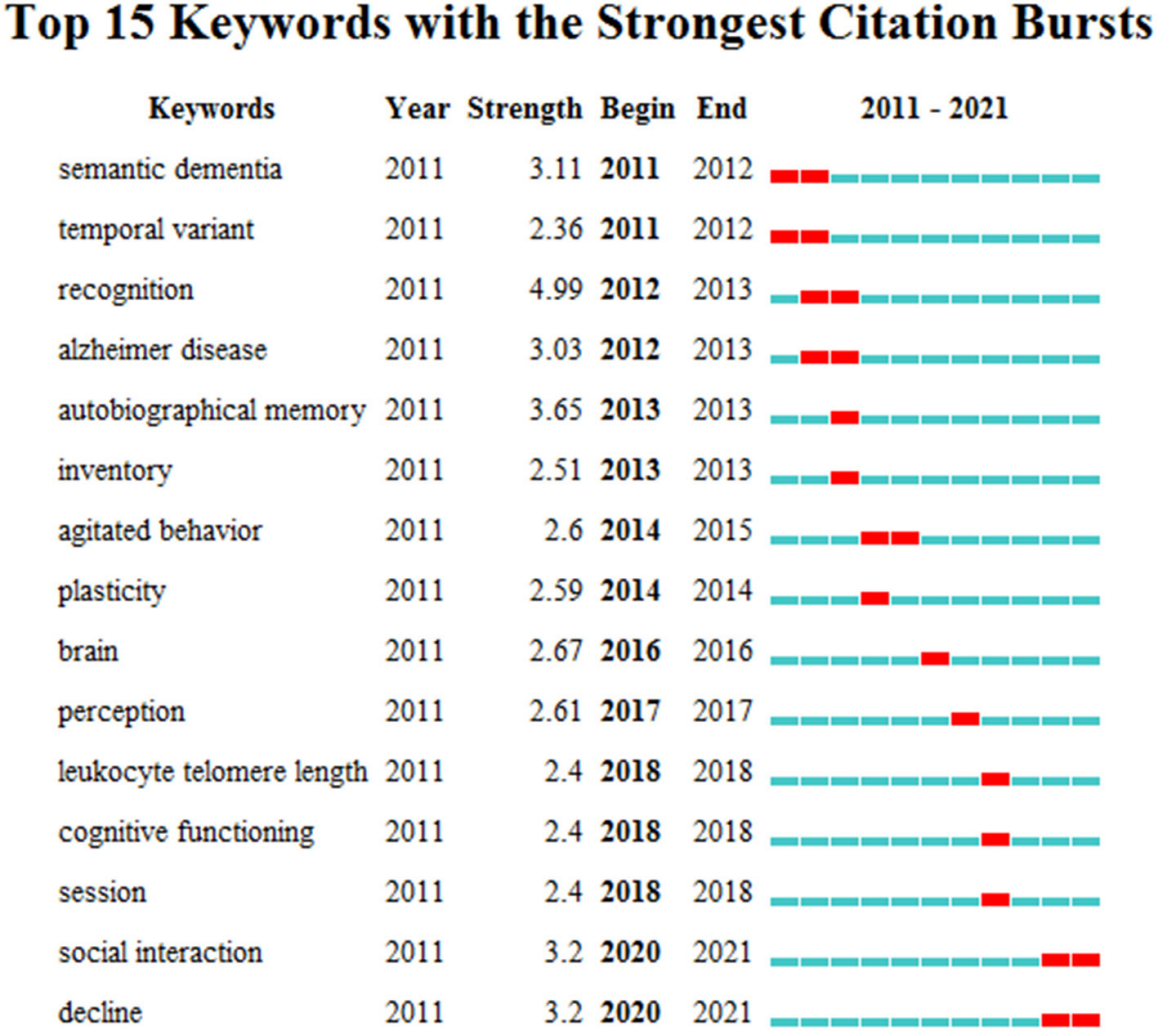
Top 15 keywords with the strongest citation bursts.

**Figure 8:**
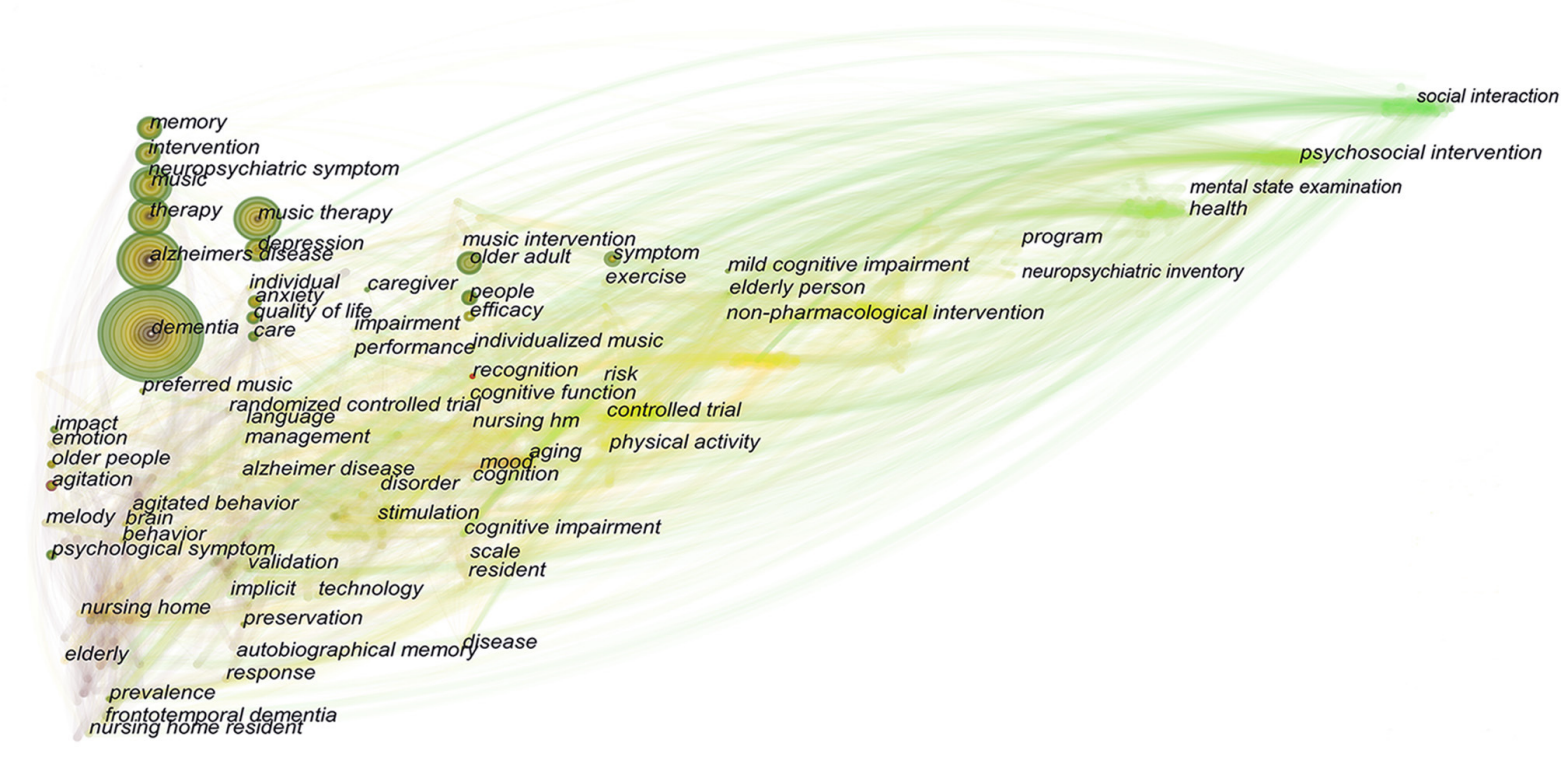
Time zone graph of keywords in articles published in the field of music therapy for dementia from 2010 to 2021.

The keyword outburst and keyword time zone map can more clearly display research hotspots and their changing trends and provide direction for follow-up research in this field. In Figure 7, 'social interaction' and 'decline' have been common keywords since 2020, and Figure 8 shows that the research field has begun to focus on the behavior of dementia patients at an early stage. Over time, research has begun to focus on the psychosocial aspects of dementia. It is worth noting that the common keyword and emergent word categories in the 2021 time zone map both include 'social interaction', indicating that current research pays more attention to social interaction and communication among dementia patients and reflects the latest research trends in this field.

### Keywords Biclustering

**Figure 9:**
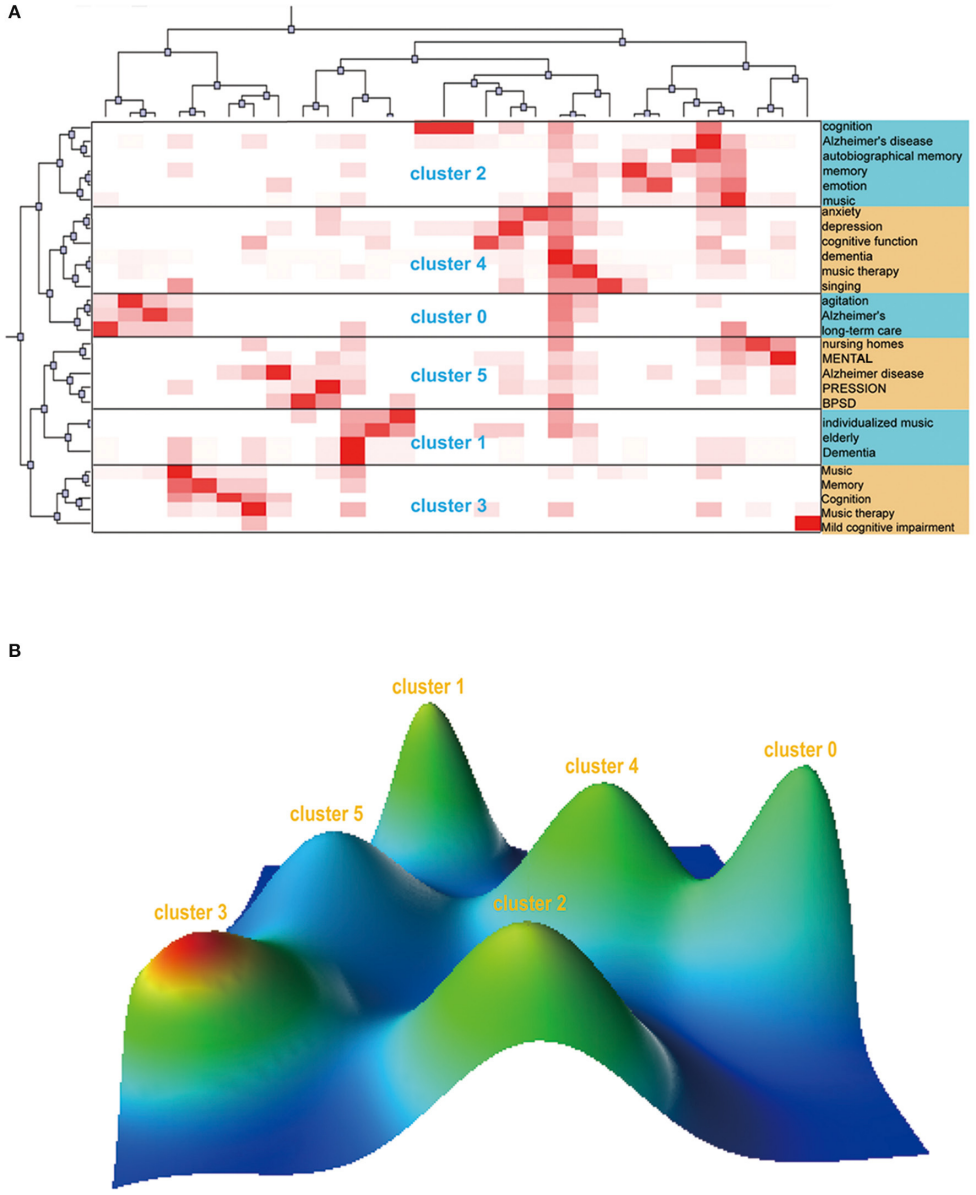
The biclustering result of highly frequent keywords and source articles in the field of music therapy for dementia from 2010 to 2021. **(A)** Matrix visualization of biclustering of 28 high-frequency keywords and source articles. **(B)** Mountain visualization of biclustering of 28 high-frequency keywords and source articles.

A total of 28 high-frequency keywords were obtained from the included studies, accounting for 5.98% of all keywords (Supplementary Table 1). The 28 high-frequency keywords were divided into 6 clusters. The visualization matrix of high-frequency keywords and source articles is shown in Figure 9A. The row labels denote high-frequency keywords, and the column labels indicate the source articles. The darker the color is, the higher the frequency of the keywords. Mountain visualization is used to verify the effect of the visualization matrix (Figure 9B), and each mountain represents a cluster. The height of the mountain is proportional to the similarity within the cluster, the volume of the mountain represents the number of high-frequency keywords in the cluster, and red indicates a low standard deviation within the cluster; otherwise, this factor is shown in blue. After analyzing high-frequency keywords in each cluster, the topic of each cluster is summarized to identify research hot spots in this field and assist subsequent research.

Cluster 0: The effect of long-term care on agitation in Alzheimer's patients.
Cluster 1: Effects of individualized music therapy on older patients with dementia.
Cluster 2: Effects of music on autobiographical memory, emotion and cognitive function in patients with Alzheimer's disease.
Cluster 3:Music therapy in the context of improving memory and cognitive impairment.
Cluster 4: Effects of music therapy on anxiety, depression and cognitive function in patients with dementia.
Cluster 5: Mental stress and BPSD symptoms in patients with Alzheimer's disease in nursing homes.

## Discussion

The analysis of annual publications, international cooperation and institutions shows that in this research area, annual publications generally show a slowly increasing trend and that the United States has the most publications and the most frequent cooperation among countries. Most research institutions in this field are universities, among which UCL has the strongest comprehensive influence. Although analysis of China's international cooperation and institutions shows that its number of publications ranks second, the central value of China is low, and the country has a lack of influential research institutions in this field. Combined with high-frequency keywords, keyword emergence and the keyword time zone map, we found that the current hotspot in this field is the psychosocial care of patients with dementia. In the time interval chosen, this research field paid more attention to the management and care of the cognitive behavior and function of patients with dementia in the early stage of the disease and explored the mechanisms potentially underlying this aspect. As time progressed, the literature also began to pay more attention to the mental health of patients. Comprehensive analysis of the six clusters found in the biclustering shows that the research hotspots in this field over the past 11 years have mainly focused on autobiographical memory, cognitive function, mental state and BPSD among dementia patients.

In China, dementia has become the fourth leading cause of death among elderly individuals after cancer, heart disease and cerebrovascular diseases (28), which places great pressure on and causes pain to dementia patients, their families, caregivers, and even society as a whole and seriously affects the quality of life of these individuals. The 2018 Guidelines for the Diagnosis and Treatment of Dementia in China indicate that non-pharmacological treatment should be the first choice for psychiatric and behavioral symptoms of dementia and that personalized treatment should be conducted to achieve the best efficacy (29). However, relevant research concerning non-pharmacological intervention remains incomplete. Some dementia patients and their families have insufficient understanding of the disease, resulting in a low attendance rate, difficult care and many problems (30) as well as a lack of exchange and cooperation with other countries and institutions in this research field in China. Therefore, in order to strengthen diagnosis and nursing and improve the health and nursing systems, China should further strengthen exchanges and cooperation with international institutions to carry out in-depth research concerning music therapy intervention for dementia patients, improve the influence of this aspect of this field, and provide more help to dementia patients.

Behavioral and psychological symptoms are the most common symptoms of dementia, with patients often experiencing anxiety, depression, sleep disturbances, apathy and irritability. The physical and mental health of the sufferer is also impaired by life disorders associated with eating difficulties and a strong sense of loneliness (31). Different types of eating difficulties caused by cognitive impairment have been reported to contribute to malnutrition in dementia patients and to increase their risk of respiratory infections (32). Several studies have shown that a lack of social communication in patients with dementia is associated not only with higher mortality but also with increased BPSD and psychological symptoms such as depression and loneliness (33, 34), among which loneliness and isolation are closely associated with dementia. One study of 12,030 participants showed that loneliness increases the risk of dementia by 40% in older adults (35), and another study of 1,547 dementia patients showed that nearly one-third of patients experience moderate social isolation and loneliness (36). Although the causal relationship between loneliness and dementia has not been clearly established (37), enhanced social communication has shown certain benefits in preventing loneliness and improving of cognitive function in dementia patients (38, 39).

Through a comprehensive analysis of the five clusters in the keywords biclustering, it can be concluded that the research hotspots in this field in the past decade have mainly focused on the autobiographical memory, cognitive function, mental state and BPSD of dementia patients. Research in this field were more focused on Alzheimer's patients, aiming to improve the mental symptoms and cognitive behavior of AD patients through music therapy, thereby improving their quality of life. As a common neurodegenerative disease, AD is the most important type of dementia. Symptoms of AD include progressive memory degeneration, language impairment, depressive symptoms and hallucinations, and the disease is accompanied by pathological changes such as cerebrovascular amyloidosis and inflammation (40). Although genetic factors are considered to be the greatest risk factor for AD, the underlying pathogenesis of AD remains unclear, and there is no effective treatment for AD (41). As a non-pharmacological therapy for the treatment of AD, the potential mechanisms of music therapy mainly include the following aspects: (1) improving patients' cognitive neural efficacy and thus affecting neuroplasticity; (2) promoting the regeneration and repair of neurons; (3) affecting hormone levels to prevent deterioration due to the disease; and (4) enhancing autobiographical memory and reducing mental symptoms (42).

At present, the daily life of dementia patients consists mainly of routine nursing care, and the caregiver plays an important role in the treatment process. Nursing homes have become a choice for many families because of the difficulty of caring for individuals with the disease. However, while this approach does relieve pressure on family members, it also entails psychological problems such as guilt and depression (43, 44). In China, more than 90% of dementia patients are cared for by family caregivers. Due to a lack of corresponding social support, family caregivers face great stress and suffer from mental and physical health problems (45). In a UK study involving 1,283 dementia patients, close to 50% of family caregivers reported feeling lonely, and close to 20% of family caregivers reported experiencing severe loneliness (46). Therefore, the psychological status of patients' family members should also be considered, since doing so can also have a positive effect on the nursing care of dementia patients.

As one of the main forms of music therapy, listening to the individual's favorite music or actively participating in musical instrument playing and singing can arouse patients' positive emotions and strongly stimulate changes in neuroplasticity. This method can play a benign role in the treatment of neurological diseases to varying degrees (47). Targeted memory music recall therapy has produced positive effects in improving the cognitive ability and happiness of elderly people (48). Although the underlying therapeutic mechanism of music therapy is unclear, such therapy has been shown to be effective and cost-effective as a nonpharmacological intervention in the treatment of mild to moderate depression in elderly individuals (49), and studies have shown that music therapy can also relieve anxiety by regulating mood (50). Some dementia patients suffer from depression, anxiety and other symptoms, so the use of music therapy has broad clinical value and can also improve the mental state of caregivers (51). In general, music therapy has certain advantages in the treatment of dementia, but there is a lack of a standardized treatment protocol for music stimulation of dementia patients, resulting in differences in treatment results. Most outcomes related to mood and spirit have been measured by self-assessment questionnaires, which may also affect the reliability of the results (52).

### Strengths and Limitations

This study is the first visualization analysis in this field based on 217 related research articles concerning music therapy intervention for dementia patients in the WoSCC database in the past 11 years. Excel, CiteSpace and gCluto were used to categorize the articles, and systematic statistics are collected concerning countries, institutions, keywords and journals, and categories were assigned according to high-frequency keywords. The co-occurrence matrix clearly and intuitively shows the research status of and trends in this field, and this study can serve as a reference for future research directions in this field.

However, this study also faces certain limitations. We only analyzed the WoSCC database, and there were only a small number of studies that met the criteria, so we could not include all relevant studies concerning the use of music therapy for dementia patients, which might entail that the literature included is incomplete and thus cannot fully reflect research hotspots and trends. Although we set the literature type to “Article” and read the full text for screening purposes, there are differences in literature quality, which may also entail certain deviations in research results.

## Conclusion

This study conducted a bibliometric and visual analysis of relevant studies concerning music therapy intervention for dementia patients in the WoSCC database, and the results indicated that the United States has the most publications and the most frequent cooperation among countries in this field and that and emerging countries are also gradually increasing. Psychological problems faced by dementia patients and the topics of quality of life, individualized music therapy, the mental state of caregivers and other related topics may be important research directions in the future. Therefore, the question of how to develop standardized research protocols and identify unified efficacy evaluation indicators should be a focus of and difficulty for future research.

## Data Availability Statement

The original contributions presented in the study are included in the article/Supplementary Material, further inquiries can be directed to the corresponding author.

## Author Contributions

SY and FZ: study conception and design. LL and DC: administrative support. ZL, YZ, and LZ: collection and assembly of data. LZ, BL, XW, and ZL: data analysis and interpretation. SY and FZ: manuscript writing. All authors final approval of manuscript and contributed to the article and approved the submitted version.

## Conflict of Interest

The authors declare that the research was conducted in the absence of any commercial or financial relationships that could be construed as a potential conflict of interest.

## Publisher's Note

All claims expressed in this article are solely those of the authors and do not necessarily represent those of their affiliated organizations, or those of the publisher, the editors and the reviewers. Any product that may be evaluated in this article, or claim that may be made by its manufacturer, is not guaranteed or endorsed by the publisher.
