# Supplementary material for: Research Hotspots and Trends in Music Therapy Intervention for Patients With Dementia: A Bibliometrics and Visual Analysis of Papers Published From 2010 to 2021
Source: Front Psychiatry. 2022 Apr 28;13:860758. doi: 10.3389/fpsyt.2022.860758 (PMC9098357; doi:10.3389/fpsyt.2022.860758)
Supplement: Supplementary file 1 [file Table_1.pdf]

Supplementary Table

NUMBER	KEYWORDS
1	dementia
2	Dementia
3	Alzheimer's disease
4	music therapy
5	music
6	Music
7	Music therapy
8	PRESSION
9	MENTIA
10	depression
11	agitation
12	Alzheimer disease
13	memory
14	cognition
15	Alzheimer's
16	cognitive function
17	individualized music
18	anxiety
19	Memory
20	BPSD
21	singing
22	Mild cognitive impairment
23	autobiographical memory
24	emotion
25	long-term care
26	Cognition
27	nursing homes
28	elderly
29	Mnemonics
30	Non-pharmacological intervention
31	intervention
32	quality of life
33	music intervention
34	Cognitive training
35	aging
36	Emotion
37	Anxiety
38	MEMORY
39	MANAGEMENT
40	older
41	playlists
42	nonpharmacological interventions
43	THERAPY

44	INDIVIDUALS
45	functional independence measure
46	SYMPTOMS
47	musical training
48	Mild cognitive
49	older adults
50	qualitative
51	musical memory
52	person-centered
53	FEASIBILITY
54	SCALE
55	DEPRESSION
56	EFFICACY
57	music stimulation
58	Japanese music
59	REHABILITATION
60	well-being
61	Activities of daily living
62	cognitive impairment
63	Aged
64	meta-analysis
65	caregiver
66	Aging
67	microswitch
68	mild cognitive impairment
69	Alzheimer's disease (AD)
70	brain rehabilitation
71	communication
72	Art therapy
73	live music
74	PEOPLE
75	elderly people
76	Rehabilitation
77	auditory
78	reward
79	Music reminiscence activity
80	behavioral
81	selfregulation
82	multisensory
83	residential
84	behavioral symptoms
85	motor
86	homes for the aged
87	human aging

88	magnetic resonance imaging
89	healthcare
90	group singing
91	immediate
92	long-term effect
93	implicit memory
94	listening to music
95	intervention design
96	incidental falls
97	individual differences
98	intuition
99	interdisciplinary collaboration
100	logopenic
101	karaoke
102	language
103	individualized music intervention
104	listening
105	meaningful
106	controlled
107	ecological momentary assessment
108	development
109	dementia-related apathy
110	dementia and cognitive
111	dance
112	daily living
113	culturally appropriate
114	crossover design
115	cross-platform
116	critical synthesis
117	creative aging
118	cooking
119	effectiveness
120	consolidation
121	concept analysis
122	community-based cognitive
123	color
124	cognitive training
125	cognitive therapy
126	cognitive reserve
127	cognitive rehabilitation
128	cognitive impairments
129	cognitive functions
130	cognitive functioning
131	cognitive dysfunction

132	group activity
133	families
134	group
135	global music approach in
136	geriatric nursing
137	frontotemporal
138	frail older adults
139	folk recreation program
140	focus group
141	feelings
142	family caregivers
143	family caregiver
144	family
145	elderly care
146	falls
147	fMRI
148	exergame
149	exercise with music
150	executive function
151	event-related
152	ethnography
153	ethnicity
154	environment
155	engagement
156	embodiment
157	elders
158	music listening
159	recall
160	senior
161	self-care
162	schizophrenia
163	scales
164	s disease
165	resting-state fMRI
166	residential care
167	reminiscence
168	recreational activities
169	recollection
170	recognition memory
171	sensor
172	rapid aging model
173	randomized trial
174	randomized controlled
175	quantitative methods and statistics

176	quality of
177	qualitative research
178	psychotropic medication
179	psychosocial interventions
180	psychometric evaluation
181	pseudo-dementia
182	preferred music
183	pragmatic clinical
184	technology
185	workforce
186	wellbeing
187	wandering
188	voxel-based
189	videogame
190	video game
191	verbal learning
192	valence
193	training
194	therapy
195	temporal lobe
196	single-case design
197	support
198	supplementary motor area
199	speech therapy
200	socioemotional
201	social psychology
202	social interaction
203	social agency
204	social
205	smartphones
206	sleep disorders
207	sleep
208	medical humanities
209	music activities
210	neurologic music therapy
211	neurocognitive disorders
212	neurobiological mechanisms of action of
213	near-infrared
214	narrative
215	musical
216	music-based therapeutic
217	music therapy and dementia
218	music program
219	music care

220	music and art
221	non-pharmacological
222	movement music therapy
223	movement
224	motor abilities
225	motivation
226	models of care
227	mnemonic
228	misidentification delusion
229	mind-body
230	methodology
231	memory complaints
232	melody
233	positivity bias
234	observation
235	positive behaviour
236	popular songs
237	pitch
238	picture colouring
239	personal psychology
240	person-centered care
241	participatory
242	outcome measure
243	older people
244	old people
245	observed emotion rating scale
246	non-pharmacological intervention
247	nursing staff
248	nursing intervention
249	nursing home residents
250	nursing facility
251	nursing care
252	nursing
253	nurse
254	nonpharmacological treatment
255	nonpharmacological
256	nonpharmacologic
257	non-pharmacological interventions
258	ACTIVATIONS
259	Front-temporal dementia
260	ICT
261	Hypertension
262	Home care
263	Healthcare

264	Group therapy
265	Group music intervention
266	Global Music Approach to Dementia
267	Gait
268	GENERATIVE MENTIA
269	GAD-7
270	Fuzzy logic
271	Frontotemporal
272	IMPACT
273	FTLD
274	Environmental sounds
275	Emotions
276	Elderly
277	Efficacy
278	EMOTION
279	EMA
280	EEG
281	Depression
282	Dementia patient
283	Dementia and cognitive disorders
284	DEMENTIA
285	DANCE
286	M-health
287	Musician
288	Music intervention
289	Mood
290	Milieu therapy
291	Mexican American
292	Memantine
293	Meditation
294	MILD
295	MENT
296	MCI
297	MARIO
298	INTERVENTIONS
299	M MENSCH
300	M DIS
301	Live music performance
302	Learning
303	Language
304	LIVE
305	LIS DC, 1992, J CLIN EXP NEUROPSYC, V14, P463, DOI
306	KNOWLEDGE
307	Japan

308	Intimate contact
309	Intervention
310	Initiative
311	Musicophilia
312	Alzheimer's dementia
313	Behavioral and psychological symptoms of dementia (BPSD)
314	Behavioral and
315	Behavior
316	BRAIN
317	BLIND
318	BEHAVIOR
319	Autobiography
320	Auditory stimulation
321	Assisted living
322	Arts therapies
323	Aptitude
324	Apathy
325	Behavioral disorders
326	Alzheimer's Dementia
327	Alzheimer&apos
328	Alzheimer Disease
329	Agitation
330	Ageing
331	Aged care
332	Affect
333	Aesthetic preferences
334	Active music therapy
335	ATTENTION
336	ANXIETY
337	ANALGESIA
338	Controlled trial
339	Chronic pain
340	Continuing care
341	Comprehensive
342	Complementary and alternative medicine
343	Companion robot
344	Communication
345	Cognitive stimuli
346	Cognitive stimulation
347	Cognitive interventions
348	Cognitive intervention
349	Cognitive function
350	Cognitive aspects

351	Binding
352	Chromogranin A
353	Cholinesterase inhibitors
354	Caregiving
355	Caregivers
356	Caregiver burden
357	Care activities
358	Canon chord progression
359	CORRELATE
360	CONVERSION
361	COMMUNITY
362	CAREGIVERS
363	Biomarkers
364	cognitive
365	agitated behaviour
366	audio-based technology
367	attention
368	assessment
369	arts-based therapies
370	arts
371	arousal
372	app
373	antipsychotics
374	antipsychotic agents
375	amyotrophic lateral sclerosis
376	amusia
377	agitation disruptiveness
378	auditory perception
379	agitated behavior
380	agitated
381	aged care
382	adaptive network-based fuzzy
383	acupuncture
384	abstraction
385	World Cafe
386	Wiimote
387	Wellbeing
388	Video-based exercise
389	Veterans
390	Vascular encephalopathy
391	VBM
392	behavioural disturbance
393	chair yoga
394	caring

395	carer
396	caregivers
397	caregiver distress
398	care practice
399	care homes
400	care giving
401	care
402	burden
403	brain
404	auditory scene analysis
405	behavioural change
406	behavioural and psychological symptoms of dementia
407	behavioural and psychological symptoms of
408	behaviour analytics
409	behaviour
410	behavioral problems
411	behavioral and psychotic symptoms of dementia
412	behavioral and psychological symptoms of dementia
413	behavioral and psychological symptoms
414	behavioral and psychiatric symptoms of dementia
415	behavior
416	awareness
417	Neuropsychiatric
418	PROGRAM
419	RCT
420	Quality of life
421	Psychosocial interventions
422	Prosody
423	Posterior cortical atrophy
424	Piano
425	Physical exercise
426	Personalized music listening
427	Personalized music
428	Person-centered care
429	Pain
430	RECOGNITION
431	PRESERVATION
432	POWER
433	PERFORMANCE
434	PAULO PR, 1978, ANN NEUROL, V4, P225
435	PAMO B, 1990, AUSTRALAS J EDUC TEC, V6, P36
436	Older people nursing
437	Older adults
438	Older

439	Nursing
440	Note density
441	Non-pharmacological interventions
442	Neuropsychiatric symptoms
443	VALIDATION
444	Senile dementia
445	Usability
446	Treatment
447	Traditional opera
448	Tonality
449	TERMINANTS
450	Subjective cognitive decline
451	Structural group
452	Song
453	Social robot
454	Severe
455	Sensor
456	RENZI E, 1962, BRAIN, V85, P665, DOI 10.1093/brain/85.4.665
457	Semantic dementia
458	STAM protocol
459	SOUTOBARRETO P, 2015, AGEING RES REV, V24, P274
460	SINGAPORE
461	S C EXPL, P33
462	Rhythmic exercise
463	Reward
464	Reminiscence therapy
465	Relationship
466	Recognition memory
467	Randomised
468	RISK
